# Muscle‐Inspired Linear Actuators by Electrochemical Oxidation of Liquid Metal Bridges

**DOI:** 10.1002/advs.202201963

**Published:** 2022-07-21

**Authors:** Jiahe Liao, Carmel Majidi

**Affiliations:** ^1^ Robotics Institute Carnegie Mellon University 5000 Forbes Ave Pittsburgh PA 15213 USA; ^2^ Mechanical Engineering Carnegie Mellon University 5000 Forbes Ave Pittsburgh PA 15213 USA

**Keywords:** bioinspired actuators, bioinspired robotics, liquid metals, microrootics, soft actuators

## Abstract

Progress in artificial muscles relies on new architectures that combine soft matter with transduction mechanisms for converting controlled stimuli into mechanical work. Liquid metal, in particular eutectic gallium–indium (EGaIn), is promising for creating an artificial muscle since it is intrinsically deformable and capable of generating significant force and shape change through low voltage stimulation. In this work, a muscle‐inspired structure for designing liquid metal actuators is presented, where EGaIn droplets are configured with copper pads to linearly contract. By theory and experiments, it is demonstrated that this design enables higher work densities and stress, making it a favorable actuator at smaller length scales. Furthermore, higher frequency (up to 5 Hz) operation is achieved by prestretching an antagonistic pair of actuators, where energy bistability enables fast‐switching actuation. Overall, this muscle‐inspired architecture shows a unique combination of low voltage operation, higher energy density at smaller scales, structural scalability, and higher frequency actuation.

## Graphical abstract

A muscle‐inspired architecture for designing liquid metal actuators is presented. Operated electrochemically at low voltages, the proposed structure enables higher work densities and stress output, making it a favorable actuator at smaller length scales. Higher frequency actuation up to 5 Hz is achieved with energy bistability by prestretching an antagonistic pair of liquid metal droplets. Multi‐cell ensemble is capable of higher force output.

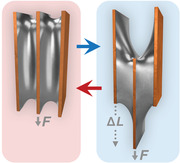

## 1 Introduction

Robotic actuators made of highly deformable materials or liquids present several design challenges since their electrical and mechanical properties, structural scalability, and dynamic performance often need to suit a robotic application.^[^ ^1^, ^2^, ^3^, ^4^, ^5^, ^6^ ^]^ For instance, dielectric elastomer actuators (DEA), while being capable of large strains (380%^[^ ^7^, ^8^, ^9^, ^10^ ^]^) and high work density (150 kJ m^−3^^[^ ^5^, ^11^, ^12^ ^]^), can only operate at high voltages (≈1 kV) and are hence limited to robots with little voltage constraints.^[^ ^13^, ^14^ ^]^ Another example is shape memory alloys (SMA), which also have high work density (10 MJ m^−3^^[^ ^5^, ^15^ ^]^) but low efficiency (3%^[^ ^5^, ^15^, ^16^ ^]^), are limited to robotic applications without significant energy constraints. Liquid metals provide a unique combination of electrical (e.g., high conductivity^[^ ^17^, ^18^ ^]^) and mechanical properties (e.g., high surface tension^[^ ^17^, ^19^, ^20^, ^21^ ^]^ in the reduced state) that are promising for building a robotic actuator. In particular, eutectic gallium–indium (EGaIn) is known to be capable of force and shape change in response to low voltage (≈1 V) inside an aqueous electrolyte solution such as sodium hydroxide and potassium hydroxide,^[^ ^19^, ^22^, ^23^, ^24^, ^25^ ^]^ which allows the EGaIn surface to reversibly oxidizes and therefore lowers the surface tension from ≈500 mN m^−1^ to near zero.^[^ ^19^, ^26^ ^]^ Surface tension‐based actuation has been demonstrated in fluid joint self‐assembly^[^ ^27^, ^28^, ^29^, ^30^, ^31^, ^32^, ^33^ ^]^ and robotic actuators.^[^ ^24^, ^25^, ^34^ ^]^ Other demonstrations with electrochemical modulation of EGaIn surface tension include droplet manipulation^[^ ^35^, ^36^, ^37^, ^38^ ^]^ and microfluidics.^[^ ^39^, ^40^, ^41^, ^42^ ^]^

Liquid metals as a practical robotic actuator instead of a mere droplet have been recently demonstrated in parallel‐plate designs,^[^ ^24^, ^25^ ^]^ where a EGaIn droplet is wetted between two parallel copper plates whose gap is energetically coupled to loading force and surface tension. This configuration, as modeled by Liao et al.,^[^ ^24^ ^]^ has a work density ≈10^2^ J m^−3^ at the mm‐scale and ≈10^5^ J m^−3^ at the µm‐scale, which makes it a favorable actuator at smaller scales. Another benefit of downscaling is the dominance of surface effects over gravity. Structural scalability of this design is limited as Shu et al.^[^ ^25^ ^]^ demonstrated that stacking at mm‐scale is only functional at no more than three layers. This is attributed to the gravitational effect that, when stacked up in layers, the aggregate weight of upper droplets eventually prevents the lower droplets from being functional. With this parallel‐plate design, dominance of surface forces at small scales is at odds with the poor structural scalability, since the latter prevents an ideal design where a prescribed liquid metal volume is split into multiple smaller droplets (with increased surface area) whose forces sum up hierarchically. This suggests a need for a new architecture to configure liquid metal droplets to achieve force and structural scalability simultaneously.

**Figure 1:**
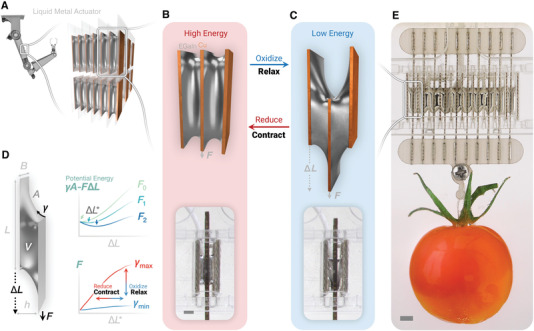
Proposed muscle‐inspired design. This liquid metal actuator design resembles the hierarchical structure of natural muscles. A) The proposed design hypothesizes a structural scalable ensemble of liquid metal actuators, B,C) each contains a EGaIn droplet bridged between two rectangular copper pads (inset images are actual actuators from Video S1, Supporting Information. Scale bar: 2 mm). D) The force *F* and length Δ*L* are coupled by the principle of minimum potential energy, where the change in surface tension γ shifts (*F*, Δ*L*) to a new equilibrium when the EGaIn surface is electrochemically oxidized or reduced at ≈1 V. E) A multi‐cell ensemble of 20 contractile units of liquid metal bridges shows capability of scaled‐up force output by the hierarchical structure (Scale bar: 5 mm).

In this work, we presented a muscle‐inspired architecture of liquid metal actuators. We hypothesized that a liquid metal actuators design where the solder joint‐like^[^ ^30^, ^43^ ^]^ liquid metal droplet wetted between two copper plates is sheared along the direction of actuator expansion and contraction, will display force scalability comparable to the parallel‐plate designs^[^ ^24^, ^25^ ^]^ and is structural scalable beyond a few droplets. When the actuator is oxidized (i.e., relaxed), the total length *L* + Δ*L* increases. When reduced, the surface energy increases and results in the overall contraction of the EGaIn bridges (**Figure** **1**B,C). Unlike the parallel‐plate design, where potential energy is asymmetrical in motion, this muscle‐inspired design has a symmetrical potential energy Π(Δ*L*) = Π(− Δ*L*), allowing for an additional capability of prestretching. By shifting one side of the plates by a nonzero λ, a pair of antagonistic liquid metal actuators can be in a tug of war with each other. We hypothesized that this enables higher frequency operation than a single cell.

We developed theoretical models to predict the shape of the actuator under different volumes, pad dimensions, surface tensions, and loading conditions (Sections S1– S5, Supporting Information). By simulating the energy minimization with surface evolver^[^ ^44^ ^]^ (Section S2, Supporting Information) and ellipse approximation (Section S3, Supporting Information), we calculated the locally minimum surface area and total potential energy accordingly. We then derived the force‐length relationship of the actuator and used it to evaluate performance, including force, displacement, and work. To experimentally test the liquid metal actuator, we manufactured mm‐scale electrode assemblies to house EGaIn droplets. We activated the actuator submerged in a potassium hydroxide (KOH) solution and recorded the response in videos. Image analysis of the actuator showed performance was compared to the theoretical prediction. The procedure was repeated with different loading forces, frequencies, and antagonistic prestretch.

## 2 Results

### 2.1 Single Cell Operations

**Figure 2:**
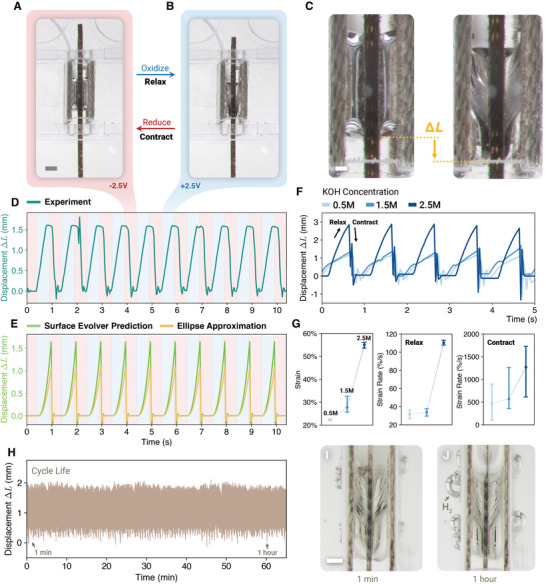
Single‐cell operations of the muscle‐inspired liquid metal actuator. A representative actuator (dimensions *L* = 5 mm, *B* = 3 mm, *h* = 1 mm) with 1 Hz activation voltage ±2.5 V in the A) reduced and B) oxidized state. Scale bar: 2 mm. The actuator is operated in a 1.5 m KOH solution. The loaded force *F* is the self weight of the actuator ≈2 mN. C,D) Actuator displacement Δ*L* is measured by video analysis of experiment where the peak‐to‐peak displacement Δ*L* ≈ 2 mm (40% strain) (Video S1, Supporting Information). E) Theoretical prediction of Δ*L* by the surface evolver and ellipse approximation (Sections S2– S4, Supporting Information) The time dependence is modeled by a spring‐mass model during reduction and by a gravitational free fall during oxidation. F) Displacements of the representative actuator with KOH electrolyte in different molar concentrations in 0.5, 1.5, and 2.5 m (Video S2, Supporting Information). G) Actuator strain output (left) and strain rate (middle and right) in different KOH concentrations based on (F). Strain rates during relaxation (middle) and contraction (right) are shown separately as the latter are typically higher by one order of magnitude. H) Cycle life test of the representative actuator in 1.5 m KOH for 1 h, where displacement is tracked over time. I) The buildup of H_2_ bubbles are compared after 1 min (I) and after 1 h (J) of operation (Video S3, Supporting Information).

We built and tested individual contractile units with dimensions *L* = 5 mm, *B* = 3 mm and *h* = 1 mm (**Figure** **2**A–C and Video S1, Supporting Information). The actuator was submerged in a 1.5 m KOH solution and was activated by an alternating voltage *V* = ±2.5 V at frequencies *f* = 1 Hz. Video analysis shows that this configuration has a maximum displacement of 2.0 mm (40% strain) when activated at 1 Hz and loading its self weight (≈2 mN, stress ≈667 Pa). The peak current draw was measured as ≈10 mA for the entire setup, which implies a power input of ≈25 mW. The actuator work output was 4.0 µ J and the work density was 266.7 J m^−3^ per liquid metal volume. By assuming all displacement occur in a periodic half‐cycle of 500 ms, we estimated a power output of 8 µ W and a power efficiency of 0.032%. The experimental measurement of Δ*L* (Figure 2D) was compared to theoretical prediction by surface evolver and ellipse approximation (Sections S2– S4, Supporting information). We note that the shape of the liquid metal bridges are modeled with the assumption that the length scale is significantly less than the capillary length (3.2 mm for EGaIn^[^ ^24^ ^]^) whereas this work implements at a scale near the capillary length. This explains the subtle drooping of the bridge in between the copper pads, which is expected to become insignificant at lower length scales. Furthermore, we tested the liquid metal actuator with different KOH concentrations (Video S2, Supporting Information), which show that higher electrolyte concentrations result in higher strains and higher strain rate (Figure 2F,G). The results suggest a nonlinear relationship between KOH molarity and actuator strain and strain rate (Figure 2G). We also conducted a cyclic testing of the actuator in a 1.5 m KOH solution for 1 h (Video S3, Supporting Information), which shows negligible degradation in actuation strain (Figure 2H), despite gradual buildup of hydrogen bubbles due to water electrolysis (Figure 2I,J). The experiments confirmed the feasibility of building a liquid metal actuator by this muscle‐inspired design.

### 2.2 Antagonistic Pairing for Higher Frequency Operations

**Figure 3:**
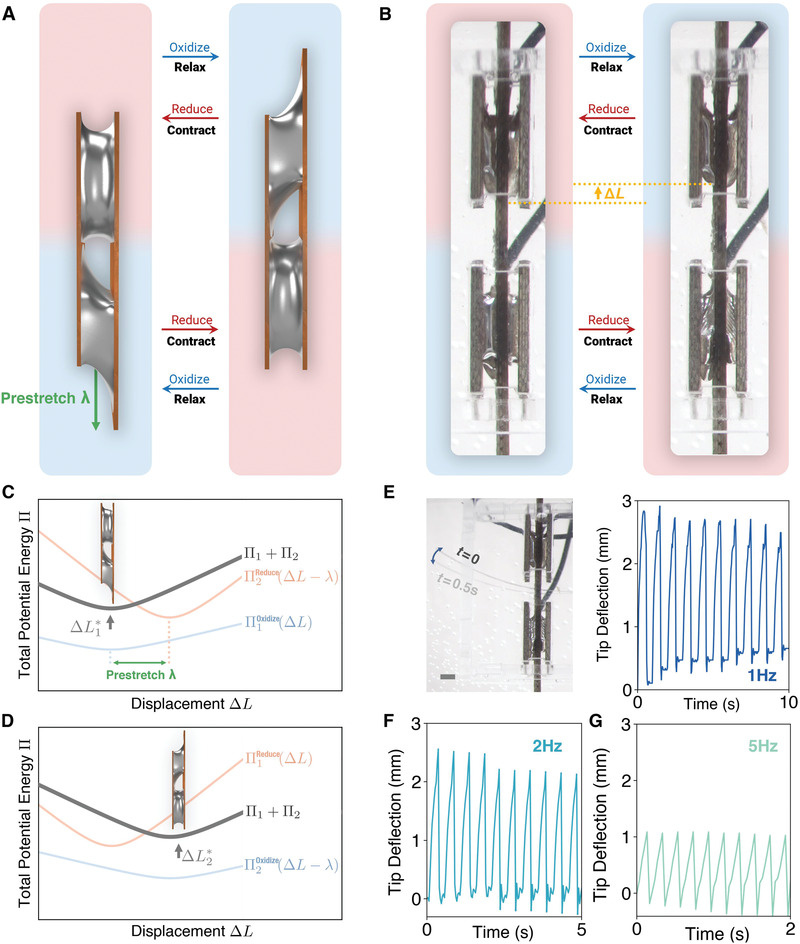
Antagonistic pairing of actuators. A) Liquid metal actuators can be paired antagonistically by prestretching one side of a pair of actuators and oxidizing the two actuators alternately. B) Actual images of an antagonistic pair of two 5 × 3 × 1 mm double‐layer actuators with a prestretch λ = 2mm, which causes a maximum displacement Δ*L*_max_ = λ. C,D) Theoretical foundation for antagonistic pairing, where bistability is introduced into the total potential energy Π_1_ + Π_2_ by adding Π_1_(Δ*L*) + Π_2_(Δ*L* − λ). When the two actuators are alternately oxidized and reduced, equilibrium length ΔL* is switched between two local minima $\Delta L_{1}^{*}$ and $\Delta L_{2}^{*}$. E) Composite image of a flexural film driven by an antagonistic pair, activated at 1 Hz (Video S4, Supporting Information). The actuation displacement (≈1 mm) is amplified by a 30 mm‐long flexural copper film, which is anchored to the actuator output 20 mm from the tip. The film bends upward at rest and therefore deflects asymmetrically due its asymmetric buckling. Scale bar: 2 mm. The plots in (E) and (G) show the tip deflection at 1, 2, and 5 Hz, with the amplitude attenuates at higher frequencies.

Given the potential energy Π(Δ*L*) of a contractile unit, we constructed antagonistic pairs of two competing actuators, where one side of the actuator is prestretched by λ (**Figure** **3**A,B). This enables a bistable actuator where the pair of actuators is alternately oxidized and reduced. This is due to the energy superposition ΣΠ(Δ*L*) = Π_1_(Δ*L*) + Π_2_(Δ*L* − λ), which creates a bistability that allows the equilibrium length Δ*L* to switch between two local minima $\Delta L_{1}^{*}$ and $\Delta L_{2}^{*}$ (Figure 3C,D). Since this configuration does not require an explicit change in force and length as in single‐cell operation (Figure 1D) to alter the energy balance, the bistable actuator can operate at higher frequency (up to 5 Hz, Figure 3E–G) by a competition between the two actuators. Video S4, Supporting Information and Figure 3E–G show that a 40% prestretched antagonistic pair (*L* = 5 mm, λ = 2 mm) caused a 20 mm flexural film to oscillate at frequencies 1, 2, and 5 Hz. This operation mode can be applied to robotic applications that require locomotion based on higher‐frequency fluidic drags, similar to the caudal fin demonstration in ref. [25] (Figure 3E).

### 2.3 Multi‐Cell Operations

**Figure 4:**
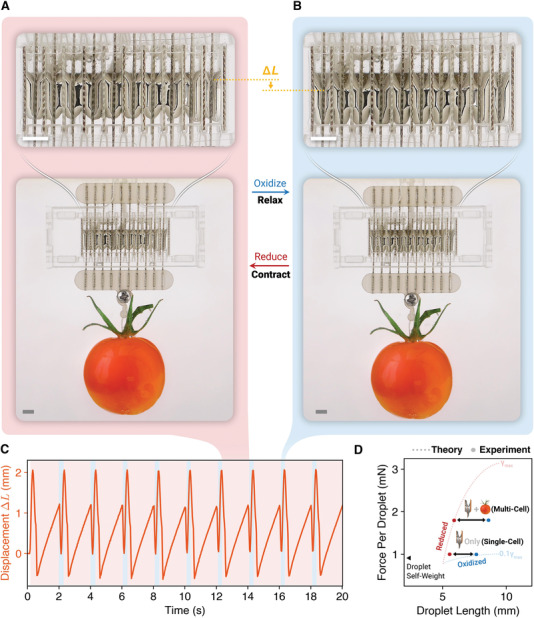
Multi‐cell operations. A,B) An ensemble of 20 liquid metal droplets (dimensions: 5 × 3 × 1 mm) lifting an adjusted cherry tomato between a reduced state (A) and an oxidized state (B) at an activation frequency of 0.5 Hz. Scale bars: 5 mm. C) Vertical displacement of the cherry tomato for ten cycles. D) Relationship between droplet elongation and dead load in the reduced and oxidized states for both the multi‐cell and single‐cell tests (Figure 2). Theoretical force–length relationships (dotted line, scaled from Figure S6, Supporting Information) are compared to the experimental results.

To demonstrate the structural scalability of this design, we built an ensemble of 20 liquid metal units with dimensions *L* = 5 mm, *B* =3 mm and *h* = 1 mm (Video S5, Supporting Information). In a 1.5 m KOH solution with voltage *V* = ±2.5V at a frequency *f* = 0.5 Hz, the actuator stack was able to lift an adjusted cherry tomato (dry weight 133 mN; buoyancy 97 mN due to the KOH solution, resulting in a net downward force 36 mN) by a displacement ≈2.7mm (**Figure** **4**A–C). We assumed that all electrodes in this configuration have equal volume and are axially and laterally symmetric, which implies the load is evenly distributed among the 20 EGaIn droplets at the same displacement. With this assumption, the net load 36 mN in this experiment (frequency: 0.5 Hz) is equivalent to 1.8 mN per droplet with equal displacement 2.7 mm, which are comparable to the single‐cell results (force: 1 mN per droplet; displacement: 2.0 mm; frequency: 1 Hz) as shown in Figure 4D. The significant oscillation ≈1 mm during contraction can be attributed to the increased loading mass *m* in a damped spring model. This demonstration confirmed stackability beyond several droplets with an overall greater force output that is relevant for robotic applications with common objects. However, since the actuator (mm‐scale) and the load object (cm‐scale) are both submerged inside the electrolyte solution, the latter is inevitably influenced by the buoyancy due to the KOH solution. This suggests further encapsulation of the liquid metal actuator, where the liquid metal droplets are packaged with a minimal amount of electrolyte (volumetric analysis in Section S8, Supporting Information), is necessary for maximizing usable force output in dry air.

### 2.4 Actuator Performance Evaluation

**Figure 5:**
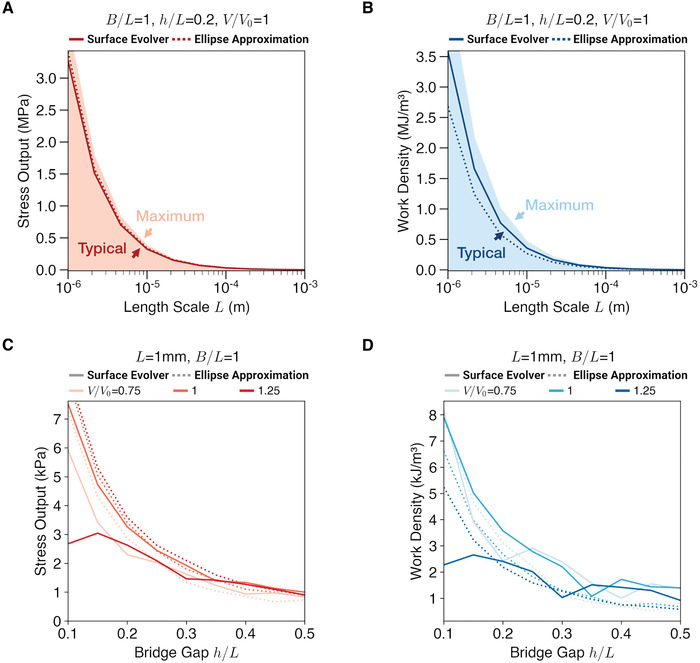
Actuator performance. Evaluation of theoretical actuator performance using surface evolver and elliptical approximation shows that A,C) stress output (force output normalized by *Bh*) increases at smaller length scale *L* and bridge gap *h*/*L*. Likewise, work density is predicted to be increasing B,D) as *L* and *h*/*L* decrease. In addition to typical (average) performance, maximum performance is reported (A and B). See Table 1; Figures S7 and S8, Supporting Information for full evaluation results.

We evaluated the theoretical performance of the liquid metal actuator by considering isometric contraction, isotonic contraction, and boundary cycle on the force–length graph *F*(ΔL*) (Figure S7, Supporting Information), where we calculate force, displacement, and work, which are normalized as stress, strain, and work density. In this work both typical and maximum performance are reported (Figure S5, Supporting Information). **Figure** **5** shows the stress output and work density for different length scales *L* of the liquid metal droplets from mm‐scale down to µ m‐scale. We assume that other elements such as the copper pads and electrolyte solution, which have volumes less than that of the liquid metal droplets by orders or magnitude (Supporting Information), are to be scaled proportionally to the liquid metal droplets and are therefore ignored in the work density calculation. We note that the aspect ratio *B*/*L*, as shown in Figure S9A–C, Supporting Information, has negligible impact on the stress output and work density and therefore the theoretical *B*/*L* = 1 results in Figure 5 can be reliably compared to the single‐cell experiment (Figure 2) where *B*/*L* = 0.6. In all cases, both surface evolver and ellipse approximation show higher stress output and work density at smaller length scales. This validates the hypothesis that the muscle‐inspired configuration shows advantages at scales *L* ≪ 1mm with higher performance due to dominance of surface tension.

### 2.5 Comparison with Previous Liquid Metal Actuator Designs

Compared to the performance evaluated in Liao et al.^[^ ^24^ ^]^ (**Table** **1**) for the parallel‐plate design (*R*_0_ = 1 mm), the muscle‐inspired design (*L*, *B*, *h* = 5,3,1 mm) in this work shows higher displacement, stress, strain, and work theoretically and experimentally, which validates the hypothesis that the muscle‐inspired design has performance comparable to the previous parallel‐plate design. This shows that the muscle‐inspired design in this work, where the liquid bridges generate force and motion by shearing, is a higher‐performance actuator than the previous parallel‐plate design in,^[^ ^24^ ^]^ where the liquid bridges generate force and motion in the normal direction to the plates.

**Table 1:** Performance comparison of the muscle‐inspired liquid metal actuators and other actuators

Actuator			Stress		Strain		Work density	
Liquid metal	Parallel plates^[^ ^24^ ^]^ (*R*_0_ = 1 mm)		637 Pa		31.5%		286.5 J m^−^3	
	Muscle inspired (this work) (*L* = 5 mm, *B* = 3 mm, *h* = 1 mm)	Theoretical	624 Pa		84.2%		684.1 J m^−3^	
		Experimental	667 Pa		40.0%		266.7 J m^−3^	
	Muscle iInspired (this work) (*L* = 2 µ m, *B* = 1.2 µ m, *h* = 1 µ m)	Theoretical	543 kPa		13.1%		757.4 kJ m^−3^	
Dielectric elastomer^[^ ^1^, ^7^, ^45^, ^46^, ^47^ ^]^			1.6 MPa		380%		150 kJ m‐^3^	
Shape memory alloys^[^ ^5^, ^15^ ^]^			200 MPa		10%		10 MJ m^−3^	
Natural muscles^[^ ^2^, ^45^, ^48^ ^]^			100 kPa		20%		8 kJ m^−3^	

### 2.6 Comparison with Natural Muscles and Other Actuators

The mm‐scale liquid metal actuator in this work (1) shows lower stress output and work density than natural muscles, dielectric elastomers, and shape memory materials. This is because liquid metals, which operate on surface forces (*F*∝*L*), do not have an advantage over other types of actuators where forces scale with cross‐sectional area (*F*∝*L* ^2^) at mm‐scale. We evaluated a hypothetical µ m‐scale liquid metal actuator at (*L*, *B*, *h* = 2,1.2,1 µ m), which showed a decrease in strain to 13.1% (since the relative gap *h*/*L* increases) and an increase in both stress output (543 kPa) and work density (757.4 kJ m^−3^), which surpass natural muscles and other existing artificial muscles in theory.

## 3 Discussion

In this work we demonstrated a muscle‐inspired design that takes lessons from the hierarchical structure of biological muscles. We used surface evolver and ellipse approximation to model the equilibrium force and shape of the liquid metal bridges and use them to evaluate actuator performance at different length scales. The numerical results suggested agreement between the two models. We demonstrate by theory and experiments that the muscle‐inspired liquid metal actuators perform with higher work densities at smaller length scales, making them a favorable actuator at smaller scales with volume constraints. Another benefit of this design is the ability of prestretching, which enables antagonistic pairing of two actuators with two bistable energy minima. This allows for higher‐frequency switching between the two states without an external force and can potentially be applied to robots which requires flexural actuation. Practically, this type of actuators can potentially be applied to applications where higher volume‐density of work output is required but which cannot afford high voltages such as the dielectric elastomer actuators.

The agreement between experimental result and theoretical prediction heavily relies on the fabrication quality, such as alignment between the pads, control of liquid metal volume, and transduction mechanism for motion observation. The material of the metallic wetting pads should be carefully selected in order to avoid reaction with the electrolyte solution. For instance, copper is known for its tendency to gradually degrade in hydroxide solutions such as NaOH and KOH where copper hydroxide Cu(OH)_2_ is formed. Although the degradation can be minimized in an ideal implementation where all the copper pads alloy with the liquid metals with no direct exposure to the KOH solution, we recommend less reactive metals such as gold and platinum for future developments. Furthermore, the requirement of an aqueous electrolyte and the hydrogen formation due to electrolysis of water remains a challenge to encapsulate the actuator. Future improvement on the design, including the electrical connection and insulation, and further study of new medium for EGaIn surface oxidation, will enable better realization of the proposed idea.

The low power efficiency 0.032% calculated from the bulk setup suggests several factors of power loss from delivering redox reactions on the liquid metal surfaces. One significant factor is the electrolysis of water, where hydrogen bubbles are periodically formed around the liquid metals as they act as the cathode during reduction. The minimum voltage in principle to electrolyze water is 1.23 V while the voltage setup in this work is ±2.5 V. Power dissipation across the KOH container, with dimensions on the order of 10 cm, can be significant with a lower concentration of KOH. Future designs where the gap between the EGaIn surfaces and the counter‐electrodes is minimized can potentially increase the energy efficiency by facilitating the ionic transport and decreasing the power loss due to ohmic loss. In Section S8, Supporting Information, we also estimated a theoretical lower bound for the electrolyte volume to be at least one order of magnitude less than that of EGaIn on the surface, which could be used as a guideline for future designs.

While this work provides a proof of concept of mm‐scale liquid metal actuators, we note that their force‐scaling advantage will only become significant once miniaturized at sub‐mm‐scales. Our models predict a stress output and work density will surpass most other existing artificial muscles at µ m‐scale, which remains a fabrication challenge. Based on the mm‐scale implementation in this work, we foresee major challenges with miniaturization through sub‐mm‐scale fabrication and self‐assembly of liquid metal droplet arrays. With fabrication at smaller scales, additional design features may be required to meet the electrical (e.g., layout for wiring, current capacity, placement of counter electrodes) and mechanical requirements (e.g., proper alignment of all electrodes to ensure symmetric placements). For depositing a large number of small‐scale liquid metal droplets, it will no longer be practical to perform wetting droplet by droplet and a more scalable self‐assembly method such as dip coating may be needed to ensure proper wetting. Ensemble behavior such as the synergistic or antagonistic interaction between a large number of droplets requires further modeling and investigation. Finally, reducing the overall volume and increasing the overall work density will require miniaturization of the supporting circuitry and electrolytic bath. Although this work has established a theoretical prediction for behavior at the small‐scale, further investigation is required to put such miniaturized systems into practice. Overall, the proposed muscle‐inspired liquid metal actuator design presents a promising small‐scale, work‐dense actuator for biologically‐inspired robotic applications.

## 4 Experimental Section

### Fabrication of Muscle‐Inspired Liquid Metal Actuators

A layered structure, which includes a laser‐cut acrylic frame and a set of FR4‐copper laminates with only copper pad areas exposed, was designed to house liquid metal bridges (Figure S10, Supporting Information). The acrylic frames were manufactured from 1/16‐inch thick acrylic sheet (McMaster‐Carr, 8560K172) cut by a CO2 50W laser cutter (Universal VLS 3.5). Customized mortise and tenon structure were built into the acrylic structure for easier assembly without adhesive or screws, which may cause unwanted chemical or electrical interactions when submerged in electrolyte solutions. Bridge gap *h* was structurally prescribed into the acrylic frame by setting the spacing between slots for the FR4 electrode sheets. Sheets of electrodes were manufactured by patterning a 1/64‐inch thick double‐sided FR4‐copper laminate (McMaster‐Carr, 8521K56) with a UV laser cutter (LPKF ProtoLaser U3; typical laser beam has a 20 µm diameter, 50 kHz frequency, and 3 W power) such that a specified number of rectangular electrodes (dimensions *L* × *B*) were exposed while the rest were insulated. The actuator structure was assembled by inserting the odd‐numbered electrode sheets into the slots in the acrylic frame, while placing even‐numbered electrode sheets freely in between the others so that they were interdigitated vertically. All copper pads were electrically connected. The assembly was then submerged in a 50% (w/v) potassium hydroxide (KOH) solution to facilitate wetting of EGaIn (75% gallium and 25% indium by weight, both from Rotometals). Droplets of EGaIn were transferred by a pipette into the space between each pair of copper pads, allowing the excess to drip by weight naturally. For simplicity we assume the bridge volume *V* ≈ *V*_0_ = *LBh* by this method. The entire assembly was then transferred to a 1.5 m KOH solution as the operating electrolyte. 1.5 m was empirically selected as the concentration to balance between redox reaction rate (faster at higher concentration) and hydrogen bubbling (slower at lower concentration).

### Single‐Cell Actuation Experiments

Electrical pulses (±2.5 V square wave) were applied at selected frequencies (1, 2, and 5 Hz) between the copper pads and the KOH solution. KOH was selected over NaOH because of its higher conductivity at the same molar concentration. The actuator was designed with weight‐hanging capability and had a self weight ≈0.2 g per electrode. The actuation response was recorded at 30 frames s^−1^ for at least 10 s in each test.

### Antagonistic Pairing Experiments

The prestretch λ in the antagonistic pairing was hard‐coded into the electrode sheets such that odd‐numbered electrodes havd larger spacings than even‐number electrodes. The role of prestretching in this type of actuators was due to the mechanism of surface tension, where the droplet relied on forces other than surface tension to deform itself in the oxidized state. In other words, in a low‐tension state, it was the external forces (e.g., loading weights) that stretch the droplet before contraction can occur. By prestretching, the pair of droplets were constantly stretched on one side and do not rely on external displacements, which was more suitable for periodic contractions at higher frequencies. The antagonistic pairs were tested following the same protocol as the single‐cell actuation where pulses were sent to each actuator specimen for video recording. A flexural copper film (width: 3 mm, length: 30 mm anchored at 20 mm from the tip) was also inserted into the moving electrode to observe the phenomenon of oscillation by bistable actuation.

### Multi‐Cell Actuation Experiments

Based on the single‐cell design, an actuator structure was created that houses an ensemble of 20 EGaIn droplets with the same dimensions (5 × 3 × 1 mm, Figure S11, Supporting Information). In order to demonstrate that the ensemble actuator was capable of generating force and motion relevant to common objects, a cherry tomato (diameter ≈23.8 mm, natural weight 46.39 g) was selected and the weight and volume were adjusted by replacing parts of the interior volume with a M3 stainless steel nut to counter the buoyancy. To waterproof the cherry tomato, the surface of the cherry tomato was coated with a transparent layer of acrylic spray paint. The overall mass of the processed cherry tomato plus the supporting structure was 13.56 g (weight 133 mN) and the buoyancy due to the KOH solution was estimated at 97 mN, which resulted in a net downward force of 36 mN. The multi‐cell actuator loaded with the processed cherry tomato was activated at 0.5 Hz for at least ten cycles.

### Image Analysis of Actuation Videos

All videos were recorded at 30 frames per second  at 4K resolution. A customized software developed in Python with scikit‐image and scikit‐video packages was used to track the dynamic positions of keypoints in every frame to calculate the actuator length changes.

## Conflict of Interest

The authors declare no conflict of interest.

## Supporting information

Supporting Information

Supplemental Video 1

Supplemental Video 2

Supplemental Video 3

Supplemental Video 4

Supplemental Video 5

## Data Availability

The data that support the findings of this study are available from the corresponding author upon reasonable request.
