# Supplementary material for: Muscle‐Inspired Linear Actuators by Electrochemical Oxidation of Liquid Metal Bridges
Source: Adv Sci (Weinh). 2022 Jul 21;9(26):2201963. doi: 10.1002/advs.202201963 (PMC9475532; doi:10.1002/advs.202201963)
Supplement: Supplementary file 1 — Supporting Information [file ADVS-9-2201963-s002.pdf]

ADVANCED SCIENCE
Open Access

Supporting Information

for *Adv. Sci.*, DOI 10.1002/advs.202201963

Muscle-Inspired Linear Actuators by Electrochemical Oxidation of Liquid Metal Bridges

*Jiahe Liao and Carmel Majidi**

Muscle-Inspired Linear Actuators
by Electrochemical Oxidation of Liquid Metal Bridges

Jiahe Liao and Carmel Majidi

Supplementary Information

1. Actuator Geometry and Kinematics

In this work we model the muscle-inspired liquid metal actuator by considering a liquid bridge wetted between two rectangular pads at a fixed gap (Fig. S1A). Let L, B be the pad dimensions and h be the bridge gap, we prescribe the volume constraint V in terms of the reference volume $V_0 = LBh$ (i.e., volume of the bounding parallelepiped). We define the free surface area A as the bridge surface area excluding the two fixed rectangles $2LB$.

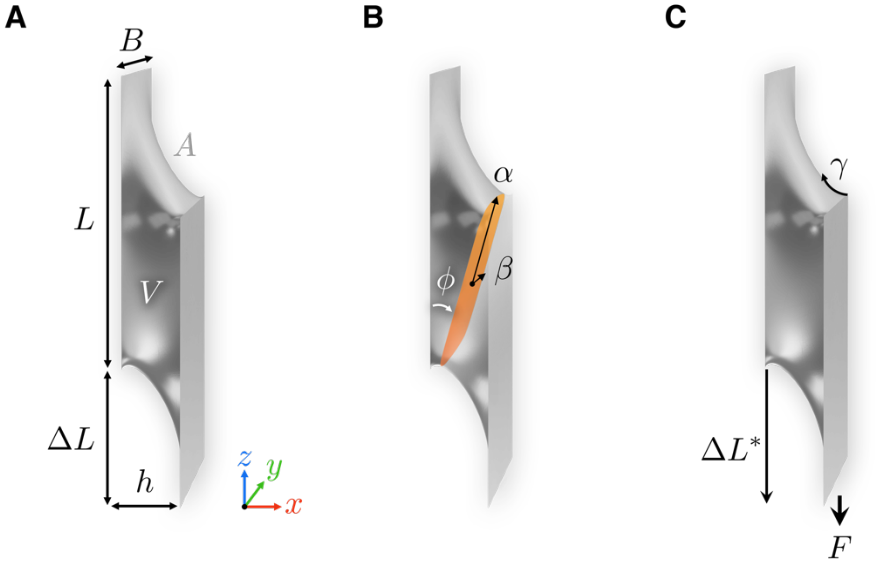

Figure S1: Geometric and kinematic parameters of the muscle-inspired liquid metal actuator. (A) A single contractile unit is a liquid metal bridge (volume V) wetted between two rectangular pads (dimensions $L \times B$) separated at a gap h. The liquid bridge shears vertically by a displacement ΔL. The interfacial area A between the liquid metal and the surrounding electrolyte changes in response to ΔL. (B) The neck of a liquid bridge is the smallest cross-sectional area that passes through the centroid. The neck shape is observed from a liquid bridge and can be parameterized by α, β, ϕ, where α and β are the neck dimensions and ϕ are the tilting angle. (C) Mechanically the geometry of the liquid metal bridge balances with the loading force F and surface tension γ, where the total potential energy $\Pi = \gamma A - F\Delta L$ is minimized.

When the liquid metal bridge has a surface tension γ, a shearing force F causes a displacement ΔL (Fig. S1C), the system has a total potential energy

$$\Pi(\Delta L;\ L, B, h, V, \gamma, F) = \gamma A(L, B, h, V, \Delta L) - F\Delta L, \quad \text{(S1)}$$

which is stationary at some equilibrium ΔL^* With prescribed parameters L, B, h, V, γ, F, local minima of Eq. (S1) depend on some arbitrary bridge shape $S(\Delta L)$ parameterized by L, B, h, V as

$$\begin{aligned}\Delta L^* &= \underset{\Delta L}{\operatorname{argmin}}\, \Pi(\Delta L) \\ &= \underset{\Delta L}{\operatorname{argmin}}\, (\gamma A(S(\Delta L;\ L, B, h, V)) - F\Delta L). \quad \text{(S2)}\end{aligned}$$

We define the *neck* as the transverse cross section (width α and depth β) of the liquid bridge that passes through the centroid and has the minimum area with a tilting angle ϕ (Fig. S1B). Intuitively, at $\Delta L = 0$, the neck is parallel to the two pads and $\phi = 0$. As the bridge is being stretched (ΔL increasing), the neck should shrink (α, β decreasing) and tilt (ϕ increasing) until the neck becomes too small and the bridge breaks.

We note that the arbitrary bridge shape S is symmetric about $\Delta L = 0$, that is,

$$S(\Delta L) = S(-\Delta L). \quad \text{(S3)}$$

It follows that the total potential energy is symmetric about $\Delta L = 0$ and $F = 0$, that is,

$$\Pi(\Delta L; F) = \Pi(-\Delta L; -F), \quad \text{(S4)}$$

and therefore the following models only consider $\Delta L \geq 0$ and $F \geq 0$.

In this work we developed two models to predict the bridge shape $S(\Delta L; L, B, h, V)$. The first is mesh energy minimization by Surface Evolver (Sec. 2) and the second is ellipse approximation (Sec. 3) by which the shape is parameterized by the neck dimensions and angle.

We ignore forces F_{gravity} due to gravity as in $F = F_{\text{surface}} + F_{\text{gravity}}$ by assuming that the liquid metal actuator operates at mm-scale at the maximum, where surface forces F_{surface} dominate. This allows us to simulate shape S independent of length scale L by reducing dimensionality in

$$A\big(S(\Delta L; L, B, h, V)\big) = L^2 A\left(S\left(\frac{\Delta L}{L}, \frac{B}{L}, \frac{h}{L}, \frac{V}{V_0}\right)\right), \quad \text{(S5)}$$

To see that this is valid at mm-scale and sub-mm-scales, we approximate the capillary length by taking the characteristic length $\lambda_c = L_c$ and solve the balance between Laplace pressure and hydrostatic pressure, that is, $\Delta P = \gamma(2/L_c) = \rho g L_c$ with density of EGaIn $\rho = 6{,}250\ \text{kg/m}^3$, for $L_c \approx 4.039\text{mm}$. This suggests that surface forces dominate when $L < L_c$.

2. Surface Evolver Simulation

We developed a program to automatically generate parallelepiped meshes with prescribed dimensionless factors $\Delta L/L$, B/L, h/L,V/V_0 (Fig. S2A) and optimize the meshes with Surface Evolver (Version 2.70, August 25, 2013) to refine them to a shape where the surface area converges to a minimum. Another benefit of normalizing all parameters by length scale L is that all computation is kept dimensionless and can be rescaled without having to optimize again. In order to avoid numerical errors and ensure the quality of meshes, after each iteration we removed the smallest 20% edges and faces with numerically zero area. We optimized each mesh for a minimum of 216 iterations (Fig. S2B-E) or until the dimensionless area A/L^2 is converged to a value where the change $\Delta A/L^2$ from the previous iteration becomes less than 10^{-6} times the initial parallelepiped surface area (Fig. S2E).

After optimization, we analyzed the mesh result and located the neck (Fig. S1B) in order to compute the dimensions (width α, depth β) and tilting angle ϕ. A set of representative results of $A(\Delta L)$, $\alpha(\Delta L)$, $\beta(\Delta L)$, and $\phi(\Delta L)$ is shown in Fig. S3. The results confirmed that with $\Delta L/L$ increasing (i.e., stretching the actuator), the surface area A/L^2 increases (Fig. S3A) while the neck dimensions α/L and β/L shrink (Fig. S3B-C) and tilting angle ϕ increases (Fig. S3D).

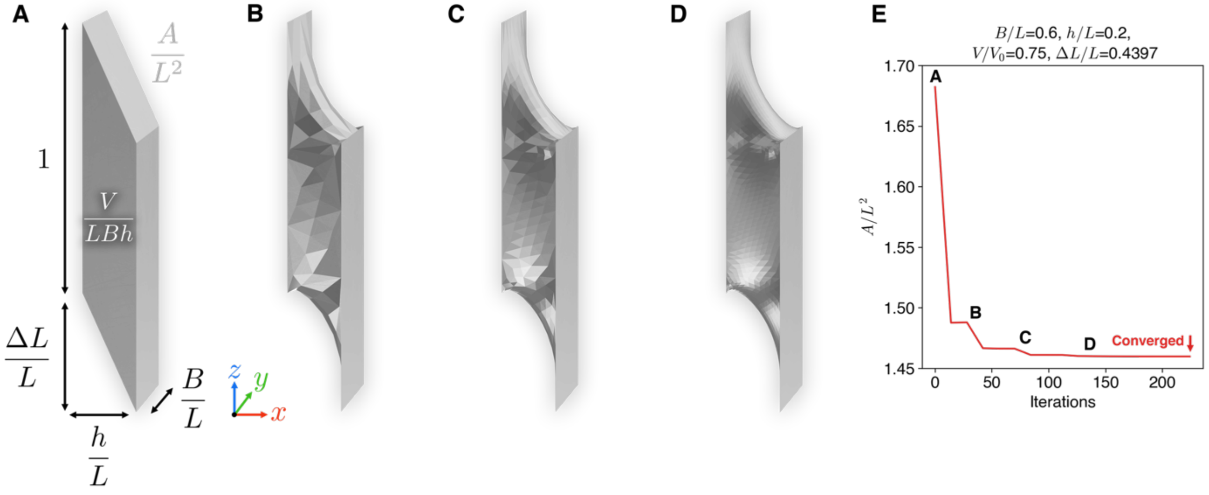

Figure S2: Surface Evolver Methodology. (A) Optimization of the shape of a liquid metal actuator is initialized by a parallelepiped with all dimensions normalized by L such that the simulation can be carried out dimensionless. Volume V is normalized by the parallelepiped volume$V_0 = LBh$. (B-D) Meshes after 30, 80, 140 iterations. (E) Convergence is determined by a relative threshold $\Delta A/L^2$.

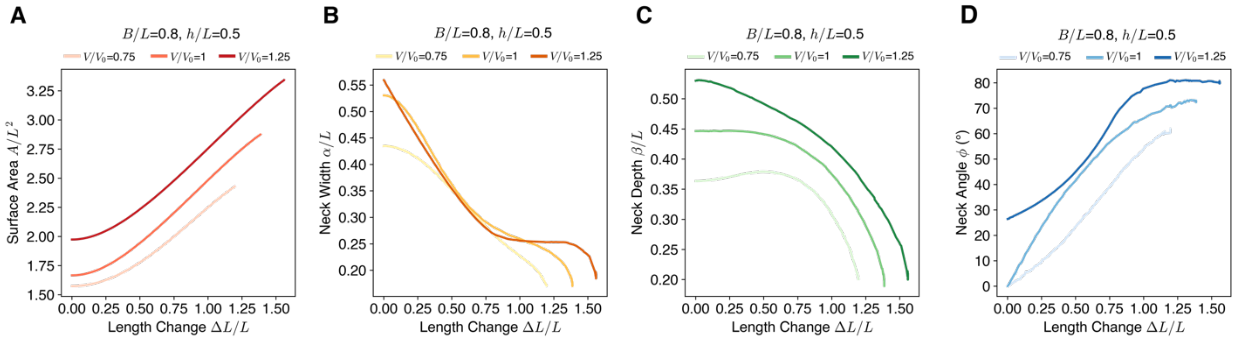

Figure S3: Surface Evolver Results. Observed geometric properties of representative actuator dimensions ($B/L = 0.8$ and $h/L = 0.5$) at different volumes (V/V_0=0.75, 1, 1.25) show that by stretching the liquid bridge with increasing $\Delta L/L$, the surface area A/L^2 increases (A) while the neck shrinks (B-C) and tilts (D).

3. Ellipse Approximation

We developed an alternative model to approximate the bridge shape by assuming the neck is always an ellipse (with semi-axes α, β) tilted at an angle ϕ, thus converting the shape approximation problem into the following two steps.

1. **Neck approximation:** Given shape parameters $(B/L,\ h/L,\ V/V_0)$, we found the least-square fit of the Surface Evolver results for (α, β, ϕ) as a function of ΔL.
2. **Surface reconstruction:** Given an ellipse (α, β, ϕ), we reconstructed a symmetric mesh that connects the two fixed rectangular pads to the neck while matching the target volume V.

The observation of an ellipse neck is shown in Fig. S5A, where the more accurate necks (Surface Evolver) tend to be more like a rectangle at smaller ΔL (Fig. S5A1-A3) and more like an oval at larger ΔL (Fig. S5A4-A5). For simplicity, we modeled all necks as an ellipse. The curve fitting methods are described as follows. Representative results of α, β, ϕ and A are shown in Fig. S5B (dotted lines) compared to the Surface Evolver results (solid lines).

Fitting Ellipse Dimensions $(\boldsymbol{\alpha}, \boldsymbol{\beta})$

We observed that the $\alpha(\Delta L)$ curves in the Surface Evolver results (solid lines in Fig. S5B) tend to have two regions separated by an inflection point around $\Delta L/L = 1$. Similar to fitting α, we observed an inflection point near $\Delta L_{\max}/2$ (solid lines in Fig. S5C). As a result, we define the following key points:

$\Delta L/L$	$\alpha(\Delta L/L;\ \boldsymbol{p})$	$\beta(\Delta L/L;\ \boldsymbol{p})$
0	$p_0 + p_1 B + p_2 (V-1)^2 + p_3 h(V-1) + p_4 hB(V-1)$	$p_0 + p_1 B + p_2 (V-1) + p_3 h(V-1) + p_4 hB(V-1)$
1	$p_0 h + p_1$	$p_0 + p_1 B + p_2 h + p_3 (V-1) + p_4 h(V-1)$
$\Delta L_{\max}/2$	-	$p_0 + p_1 B + p_2 h + p_3 (V-1) + p_4 h(V-1)$
$\Delta L_{\max}$	$p_0 + p_1 B + p_2 h + p_3 (V-1) + p_4 h(V-1)$	-

where $\boldsymbol{p}$ in each case is a set of parameters $(p_0, p_1, \ldots)$ to be fit in the least-squares sense.

With these points we fit the overall curves $\alpha(\Delta L)$ and $\beta(\Delta L)$ as

$$\alpha(\Delta L/L;\boldsymbol{p}) = \begin{cases} \alpha(0) + (\alpha(1) - \alpha(0)) \min(1, \Delta L + p_0 V + p_1) & \text{if } \Delta L \leq L \\ \alpha(1) + (\alpha(\Delta L_{\max}) - \alpha(1)) \frac{\Delta L - 1}{\Delta L_{\max} - 1} & \text{if } \Delta L > L \end{cases} \tag{S6}$$

$$\beta(\Delta L/L;\boldsymbol{p}) = \begin{cases} \beta(0) + (\beta(1) - \beta(0)) \left(\frac{\Delta L}{\Delta L_{\max}/2}\right)^2 & \text{if } \Delta L \leq L \\ \beta(1) + (\beta(\Delta L_{\max}) - \beta(1)) \left(\frac{\Delta L - \Delta L_{\max}/2}{\Delta L_{\max}/2}\right)^2 & \text{if } \Delta L > L \end{cases}. \tag{S7}$$

Fitting Ellipse Angle $\boldsymbol{\phi}$

The neck tilting angle ϕ generally increases from 0° at $\Delta L = 0$. By observation, volume V and gap h affect the growth of ϕ, which increases more aggressively with higher V and higher h. As a result, we fit the overall curve as

$$\phi(\Delta L/L;\ \boldsymbol{p}) = \min(90°,\ p_0 V\ \Delta L^{p_1 V + p_2 h + p_3})\ . \tag{S8}$$

Surface Reconstruction

To recreate a mesh that represents a shape connecting the ellipse and the fixed pads, we discretized the ellipse (α, β, ϕ) and two rectangles (L, B) into $N = 50$ points each. We then generated pair-wise parabolas between the ellipse and a rectangle with a correction factor k ($k = 1$ means straight lines, $k > 1$ convex, $k < 1$ concave). We then find the optimal k that matches the target volume V by

$$\underset{k}{\text{Minimize}} \left(V - \text{Volume}\left(S_k(\Delta L)\right)\right)^2 \tag{S9}$$

where S_k is the shape constructed from all $N = 50$ curves between the ellipse and the two rectangles by a correction factor k. Fig. S4 shows a representative mesh constructed by this method

compared to the optimized Surface Evolver mesh. Representative curves of surface area by ellipse approximation is shown in Fig. S5E.

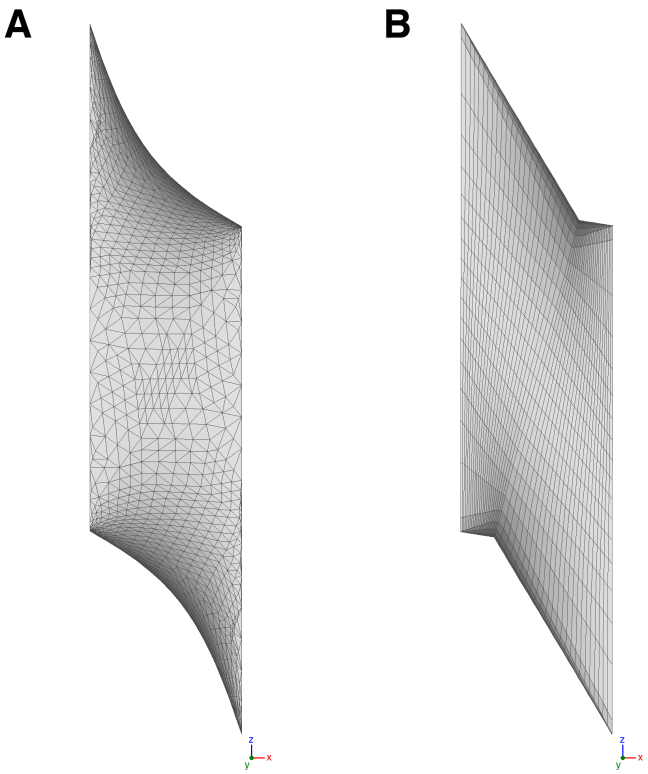

Figure S4: Surface reconstruction by ellipse approximation. Representative meshes of a sheared liquid bridge ($B/L = 0.6$, $h/L = 0.3$, $\Delta L/L = 0.4$, $V/V\_0 = 0.75$) generated by (A) Surface Evolver and (B) ellipse approximation.

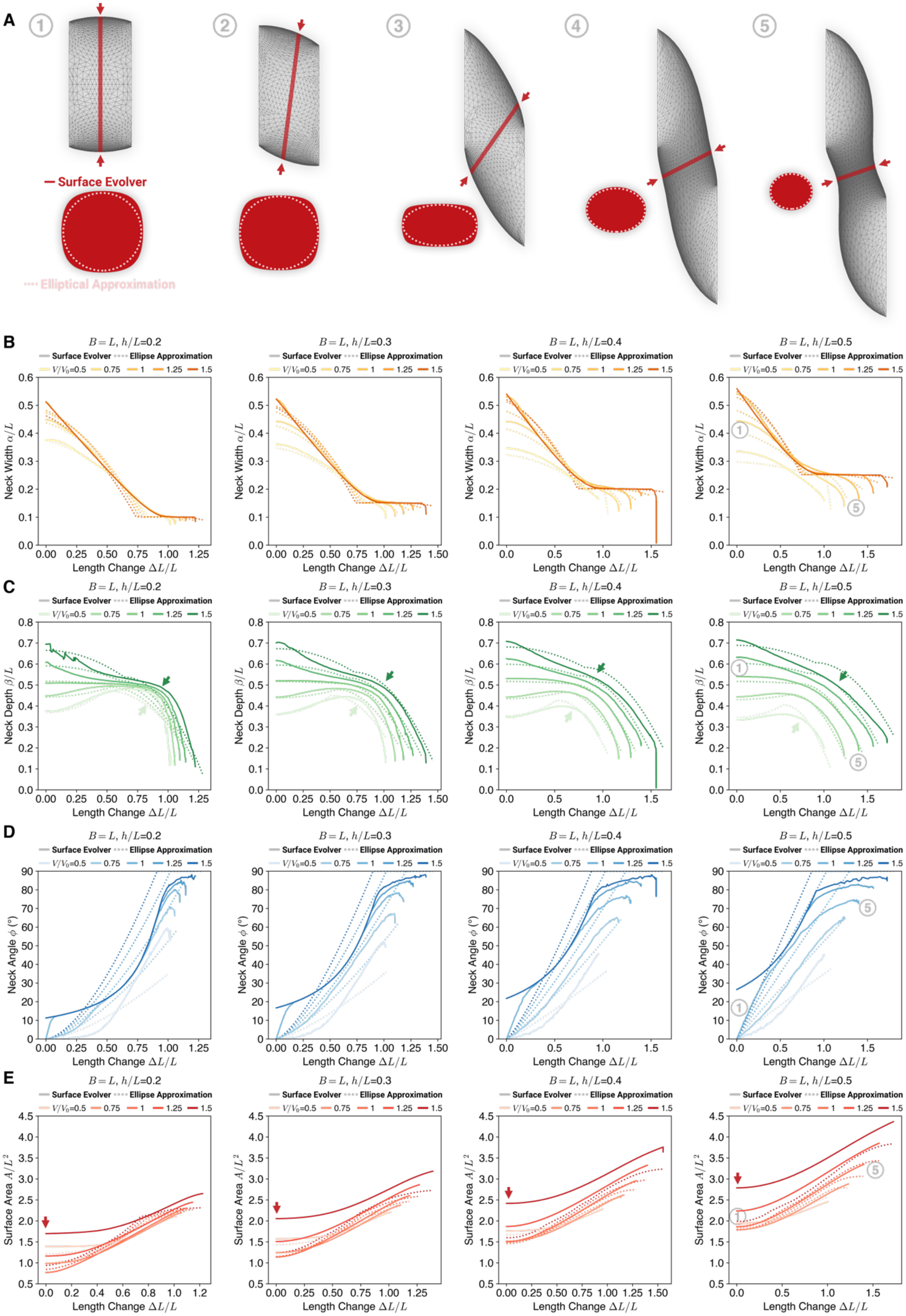

Figure S5. Elliptical approximation. (A)Shape of a liquid bridge captured by estimating the neck cross-section (red) as an ellipse (dotted) parameterized by (α, β, ϕ) (B-D) Compare the elliptical parameters fitted from the Surface Evolver data. (E) Approximated surface areas show similar trends as Surface Evolver.

4. Force-Length Relationship

The muscle-inspired liquid metal actuator operates on the principle that by controlling surface tension γ, we can shift the equilibrium force F and length ΔL^* (Fig. S2). The understanding of the mechanical behavior relies on the force-length curves $\mathrm{F}(\Delta L^*)$.

To calculate the equilibrium length ΔL^* from each of the two models, we generate the energy curves $\Pi(\Delta L)$ (Eq. S1) and locate the first local minimum for a given F (Fig. S6A). The results show that the actuator is capable of being stretched until either the shape is geometrically impossible due to a zero neck (i.e., $\alpha \sim 0$ or $\beta \sim 0$) or energy $\Pi(\Delta L)$ loses convexity and has no local minima, which has the physical meaning of bridge separation because no finite ΔL can balance Eq. S1 in any way.

With equilibrium length ΔL^* calculated for every force F, we constructed the force-length curves $F(\Delta L^*)$ with a reduced surface tension $\gamma_{\max}$ and an oxidized $\gamma_{\min} = 0.1\,\gamma_{\max}$. Fig. S6B shows a set of representative $F(\Delta L^*)$ curves, which mark the operation boundary of the liquid metal actuator. By electrochemically oxidizing and reducing the EGaIn surface, the force-length coupling $(F, \Delta L^*)$ can be switched between the two curves.

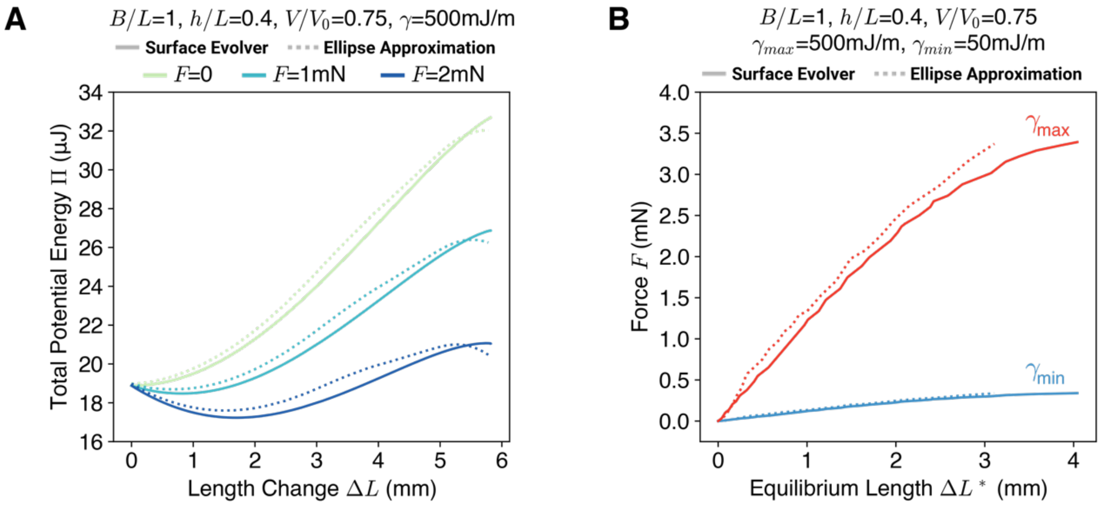

Figure S6. Mechanical behavior of the liquid metal actuator. (A) Increasing force F shifts the equilibrium length change ΔL^* at which the total potential energy $\Pi = \gamma A - F\Delta L$ is minimized. The actuator is capable of being stretched until either the shape is geometrically impossible due to a zero neck or energy $\Pi(\Delta L)$ loses convexity and has no local minima. (B) A representative force-length relationship of a liquid metal actuator. Coupling of force F and equilibrium length ΔL^* is switchable between a maximum surface tension $\gamma_{\max}$, which occurs when the liquid metal is reduced, and an oxidized low surface tension $\gamma_{\min}$ defined as $0.1\gamma_{\max}$ in this work.

5. Performance Evaluation

We evaluated the theoretical performance of the liquid metal actuator by considering one of the three operations on a force-length relationship graph.

**Isometric contraction**	Changing F between two forces while fixing ΔL^* (Fig. S6A)
**Isotonic contraction**	Changing ΔL^* between two forces while fixing F (Fig. S6A)
**Boundary cycle**	Moving the coupling $(F, \Delta L^*)$ counter-clockwise around the operational boundaries. The enclosed area is the averaged work across all possible $(F, \Delta L^*)$ (Fig. S6B)

With the isometric contraction, isotonic contraction, and boundary cycle, we define the *typical* (average) metrics as follows. We also report the *maximum* values by each metric.

$$\textbf{Force} = (\text{Enclosed Area})/\Delta L^*_{\text{max}} \rightarrow \textbf{Stress} = \text{Force}/(Bh)$$

$$\textbf{Displacement} = (\text{Enclosed Area})/F_{\text{max}} \rightarrow \textbf{Strain} = \text{Displacement}/L$$

$$\textbf{Work} = (\text{Enclosed Area}) \rightarrow \textbf{Work Density} = \text{Work}/V$$

The performance calculated from the Surface Evolver (solid lines) and ellipse approximation (dotted lines) results are shown in Fig. S8. Actuator Performance at different aspect ratios and bridge gaps are shown in Fig. S9.

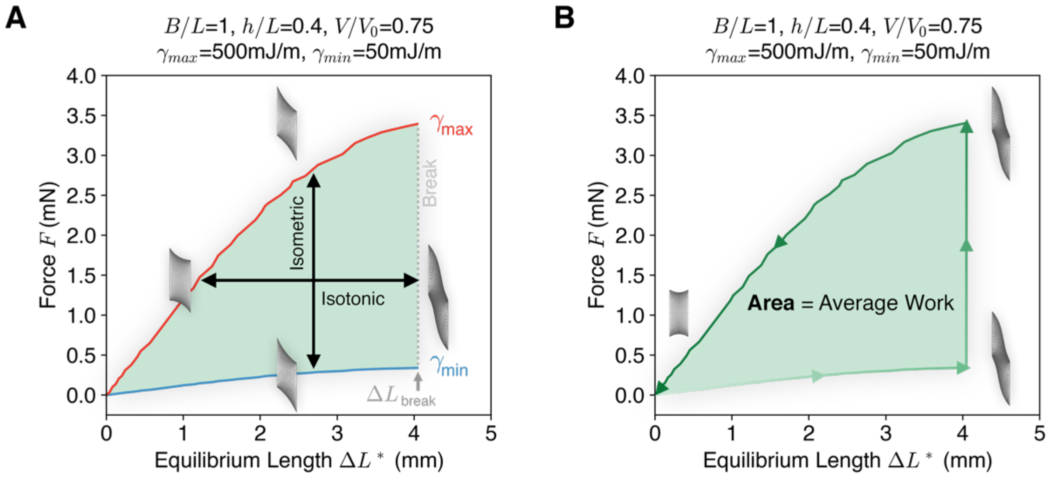

Figure S7. Performance evaluation methodology. (A) Force and displacement output from the liquid metal actuator can be measured by performing isometric contraction (changing F while fixing ΔL^*) and isotonic contraction (changing ΔL^* while fixing F). Maximum force and displacement output can be found by measuring across the full ranges of F and ΔL^*. (B) The area enclosed by the boundary motions gives the average work output across the entire range of motion.

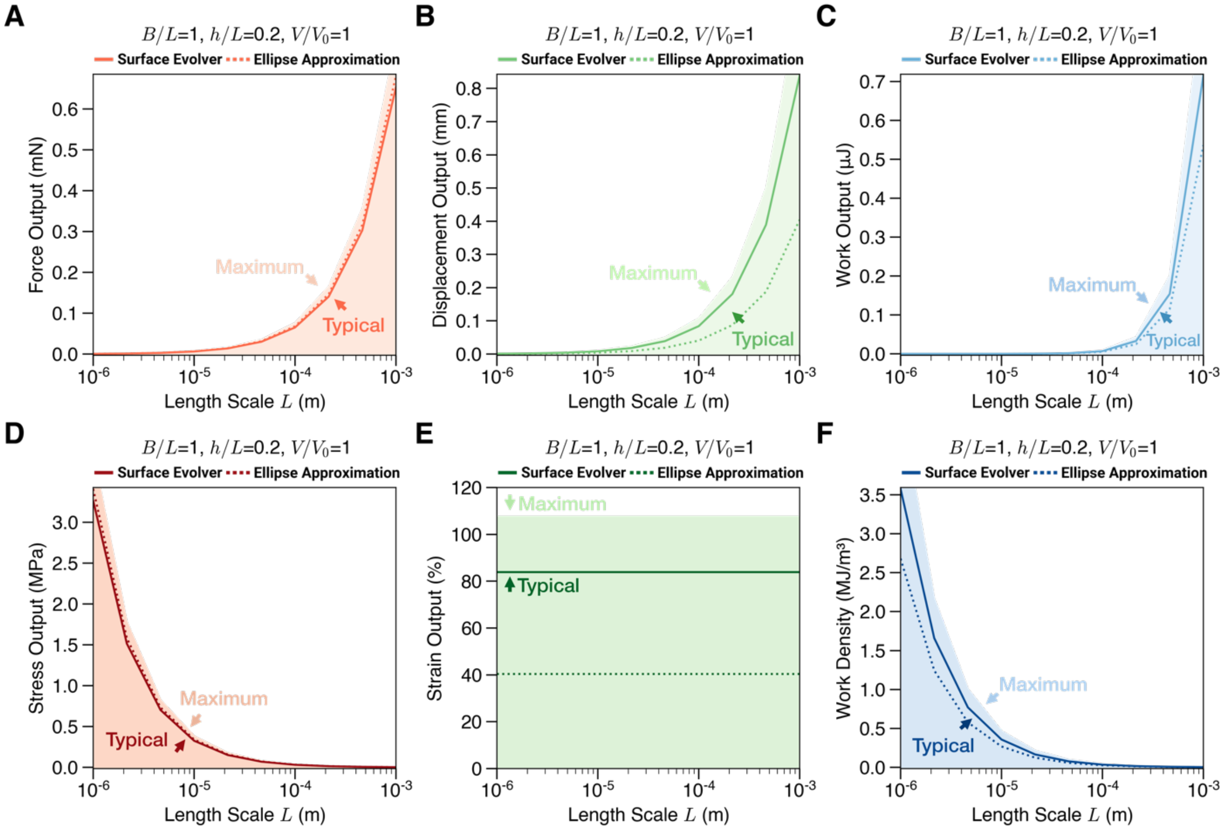

Figure S8. Actuator performance at different length scales. The performance calculated from the Surface Evolver (solid lines) and ellipse approximation (dotted lines) results are shown. Typical (i.e. average) and maximum values are reported. Force, displacement, and work output (A-C) are non-normalized. (D) Stress, defined as force normalized by Bh, increases at smaller L. (E) Strain is scale-invariant. (F) Work density, defined as the volume-density of work, increases at a smaller L.}

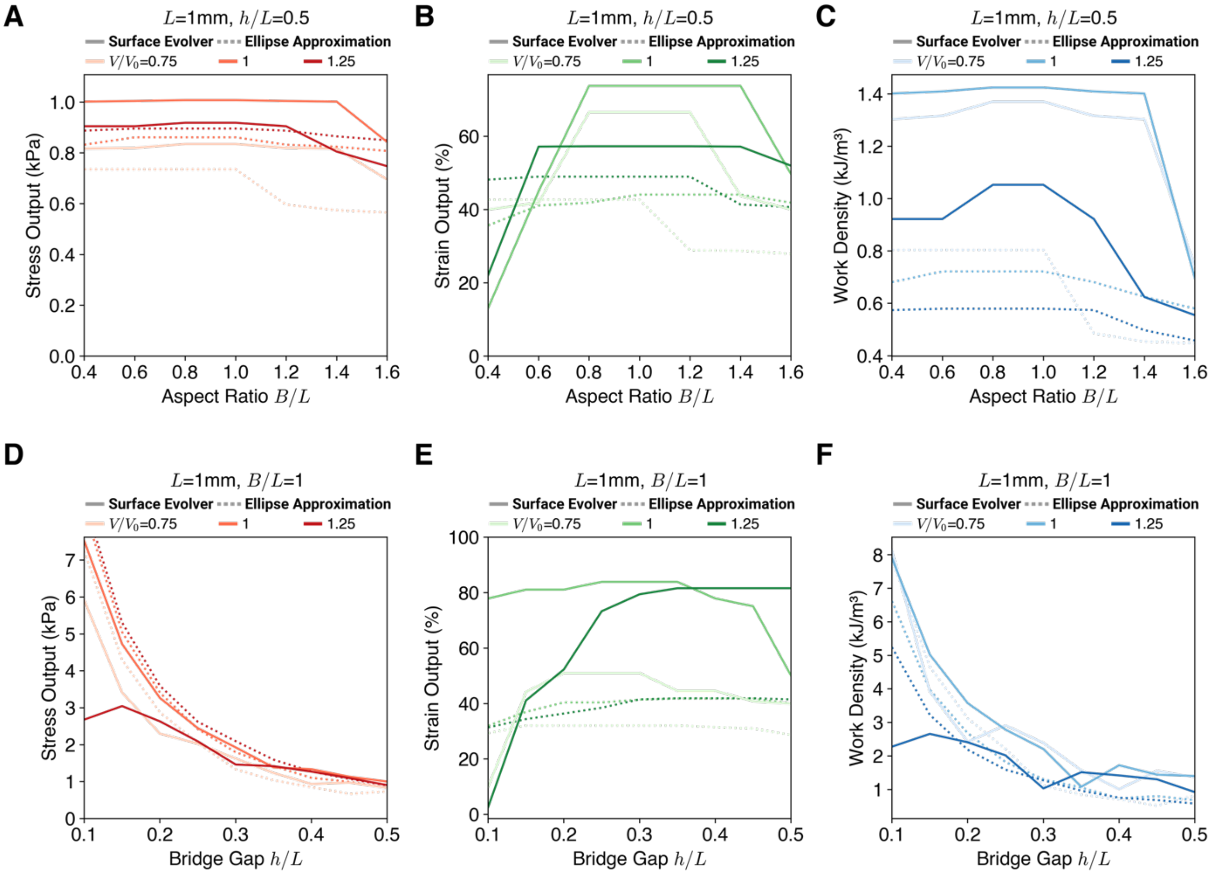

Figure S9. Actuator performance at different aspect ratios and bridge gaps. Across different volumes (V/V_0=0.75,1,1.25) the performance (stress, strain, and work density) displays insignificant change with respect to aspect ratio B/L (A-C), which indicates that aspect ratio has negligible influence on actuator performance. (D) and (E) show that bridge gap h/L has a direct impact on stress output and work density, which generally increase at a smaller h.}

6. Fabrication and Experiment of Single-Cell Actuators

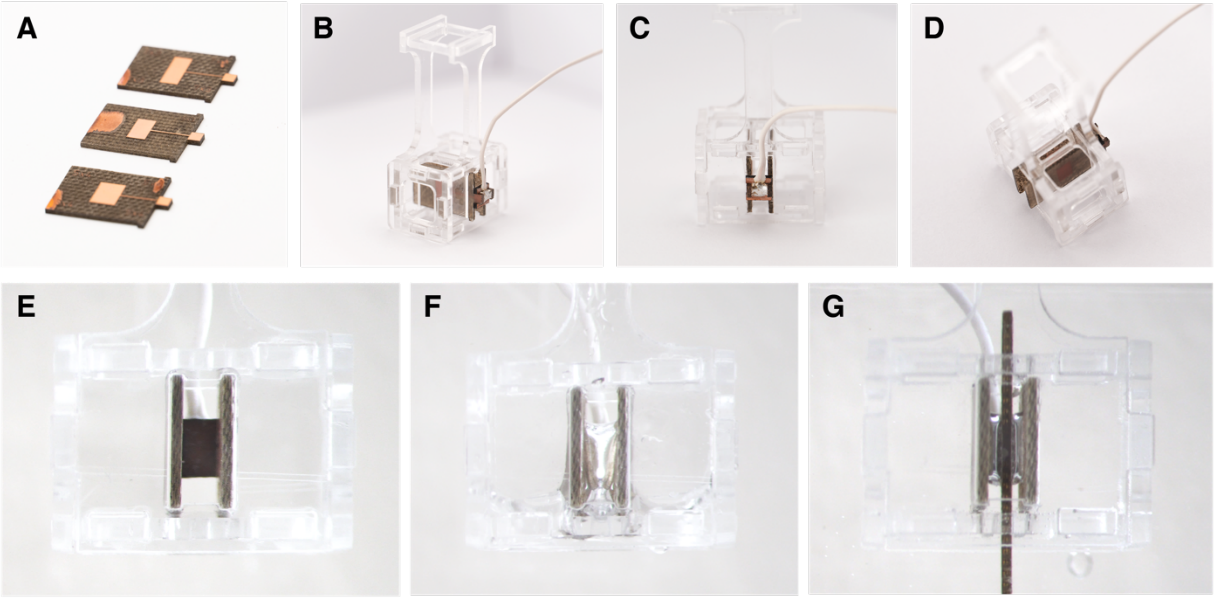

Figure S10. Fabrication of the muscle-inspired liquid metal actuators. (A) Electrode sheets were made from FR4-copper laminates, which were cut by a UV laser (LPKF ProtoLaser U3). (B-D) Actuator structures were assembled by inserting the electrode sheets into a customized acrylic frame and ensuring all the copper pads are electrically connected. (E) Actuator structure without EGaIn bridges. (F) When transferred in between the copper pads, an EGaIn bridge naturally formed and drooped in the absence of KOH solution. (G) Complete actuator assembly with two EGaIn bridges in KOH solution.

7. Fabrication and Experiment of Multi-Cell Actuators

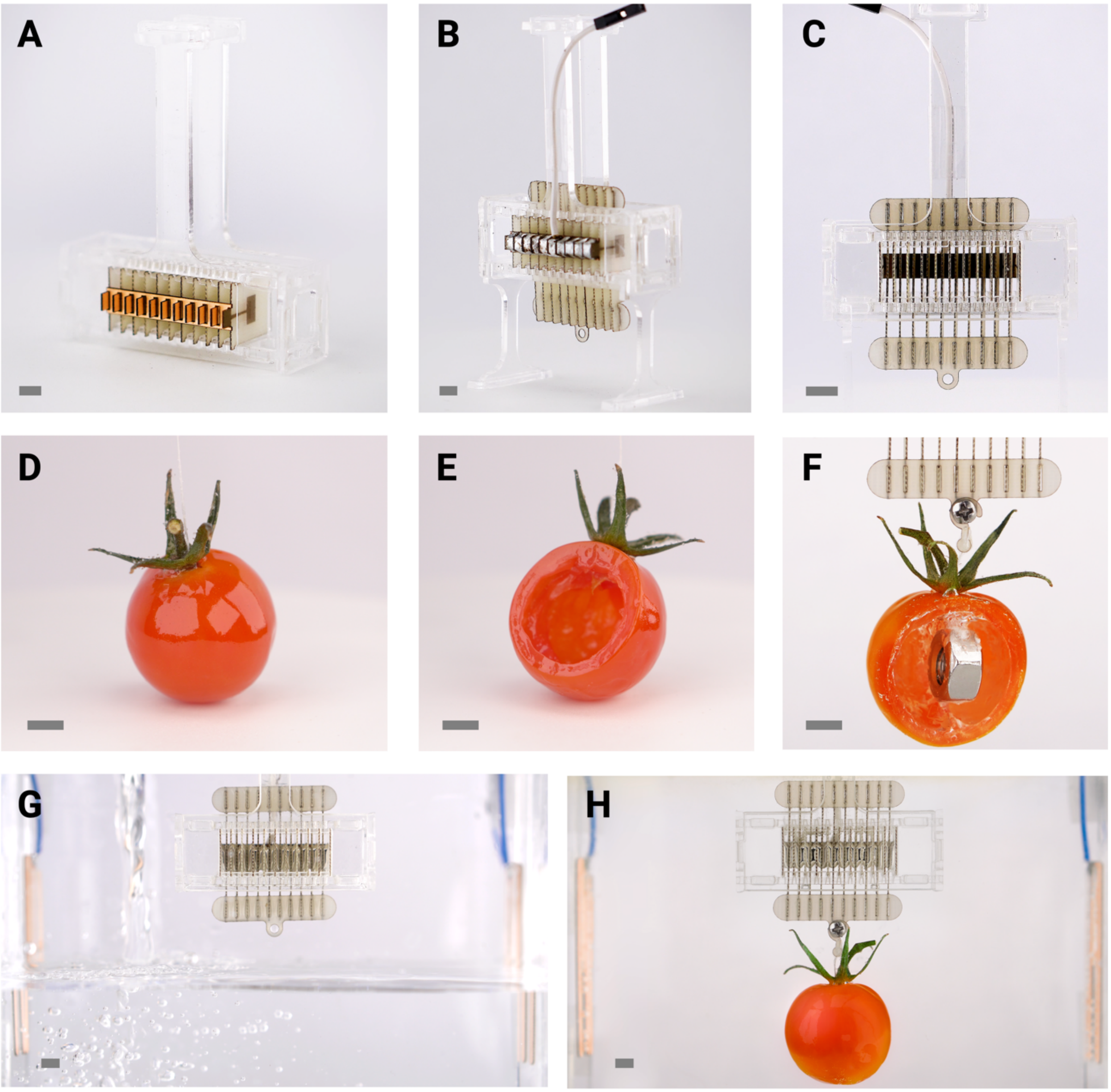

Figure S11. Fabrication and experiment of the multi-cell liquid metal actuators. (A) Ensemble design that is capable of housing 20 droplets (dimensions: 5x3x1 mm). The copper interconnect across all FR4-copper electrodes is not yet soldered. (B) Full assembly of the multi-cell actuator with the middle electrodes inserted and the copper interconnect soldered to a wire. The two legs at the bottom comprise a temporary actuator stand, which is not part of the actuator structure. (C) Image of evenly separated electrodes before wetted with liquid metals. (D-E) A cherry tomato with partial volume replaced with a M3 stainless steel nut (F) to counter the buoyancy. The overall mass is 13.56 g. (G) The ensemble actuator in a tank being filled with a 1.5M KOH solution. (H) Complete setup with the multi-cell actuator fixed with the processed cherry tomato. All scale bars: 5 mm.

8. Volumetric Analysis of Liquid Metals and Electrolytes

In this section we analyze the volumes of liquid metals and electrolytes involved in the following surface reactions:

$$[Ga(OH)_4]^- + H^+ \longrightarrow Ga(OH)_3 + H_2O$$

$$2Ga(OH)_3 \longrightarrow Ga_2O_3 + 3H_2O$$

where the hydrogen ions H^+ are provided by the electrolysis of water,

$$2H_2O \longrightarrow 4H^+ + O_2 + 4e^-$$

and the gallate ions $[Ga(OH)_4]^-$ are provided by dissolution the gallium into the electrolyte,

$$2Ga + 2KOH + 6H_2O \rightarrow 2K\,[Ga(OH)_4] + 3H_2$$

From these reactions, we can write the mass ratio of gallium versus the electrolyte components:

$$\text{Mass ratio of Ga to} \begin{array}{ll} H_2O & = 1 : 0.775 \\ KOH & = 1 : 0.805 \end{array}$$

Assume the EGaIn composition (75% Ga + 25% In by weight) and 1.5M KOH solution are used, we can rewrite the mass ratio as:

$$\text{Mass ratio of EGaIn : KOH} \quad = \quad 1 : 1.037$$

Theoretical Lower Bound of Volumes

Using the densities of EGaIn (6250 kg/m^3) and KOH (2120 kg/m^3, 1091 kg/m^3 as a 1.5M solution in water), we can write the volume ratio

$$\text{Volume ratio of EGaIn : KOH} = 1 : 0.099$$

which roughly suggests that the volume of KOH required is **at least 1 order of magnitude less than the EGaIn** on the surface, invariant of the length scales.

Practically, the electrolyte volume is dictated more by the actuator structure, which in this work is a tank of KOH bath. More sophisticated designs are necessary for the actual electrolyte to be near the theoretical lower bound.

Description of Supplementary Videos

Video 1: Single Cell Operations

An individual contractile unit with dimensions L=5mm, B=3mm and h=1mm is demonstrated in this video. The actuator is submerged in a 1.5M KOH solution and is activated by an alternative voltage $V = \pm 2.5\text{V}$ at frequencies f=1 Hz. The force applied to the actuator is the weight of the middle electrode plus the two EGaIn bridges. The video shows the 1 Hz actuation with a maximum displacement of 2.0 mm (40% strain), which corresponds to a work output of 4.01 μJ and a work density 267.2 J/m^3.

Video 2: Operation in Different Electrolyte Concentrations

The effect of three different electrolyte concentrations of KOH solution at 0.5M, 1.5M, and 2.5M on the actuator displacement is demonstrated in this video. All actuators have the same dimensions L=5mm, B=3mm and h=1mm and are activated at a frequency of 1 Hz. The video shows that higher concentrations result in higher strains and higher strain rates.

Video 3: Actuator Cycle Life

A cyclic testing of a liquid metal actuator with dimensions L=5mm, B=3mm and h=1mm for 1 hour is demonstrated in the video. The actuator is activated at 1 Hz in a 1.5M KOH solution. The video shows a 1-hour progression of cyclic activation with no visible degradation in actuation strain, despite the gradual buildup of hydrogen bubbles due to water electrolysis.

Video 4: Antagonistic Pairing for Higher Frequency Operations

An antagonistic pair of liquid metal actuators with L=5mm, B=3mm and h=1mm is demonstrated at various activation frequencies. First, the pair of two actuators submerged in a 1.5M KOH solution are oxidized and reduced alternately at 1 Hz, which causes rapid switching of displacement between two bistable local minima. Second, a flexural copper film is attached to the actuator to demonstrate higher-frequency operations, where the tip deflections activated at frequencies from 0.5 Hz to 5 Hz (by 0.5 Hz increment) are shown in the video.

Video 5: Multi-Cell Operation

An ensemble of 20 liquid metal contractile units, each with dimensions L=5mm, B=3mm and h=1mm, is demonstrated in this video. The multi-cell liquid metal actuator is submerged in a 1.5M KOH solution, activated at 0.5 Hz, and loaded with a cherry tomato with partial volume replaced with a M3 stainless steel nut to counter the buoyancy. The overall mass of the processed cherry tomato plus the supporting structure is 13.56 g (weight 133 mN) and the buoyancy due to the KOH solution is estimated at 97 mN, which results in a net downward force of 36 mN. The first part of the video shows 10 cycles of relaxation and contraction, where the bolt at the upper tip of the cherry tomato undergoes a periodic displacement of 2.7 mm. The second part of the video shows the same 10 cycles with the 20 EGaIn bridges magnified for a closeup view to the shape transition of the liquid metal surfaces.
